# Video Laryngoscopy vs. Direct Laryngoscopy in Teaching Neonatal Endotracheal Intubation: A Simulation-Based Study

**DOI:** 10.7759/cureus.962

**Published:** 2017-01-06

**Authors:** Srikumar Nair, Eric J Thomas, Lakshmi Katakam

**Affiliations:** 1 Neonatology, Children's Hospital Los Angeles; 2 Internal Medicine, McGovern Medical School, The University of Texas Medical Health Science Center at Houston; 3 Neonatology, Texas Children's Hospital

**Keywords:** neonatal intubation, simulation, video laryngoscope

## Abstract

**Background:**

Neonatal endotracheal intubation is a life-saving procedural skill where best practices have been developed from expert opinion. Few empirical studies have examined how this skill should be taught.

**Objective:**

To determine whether a video laryngoscope (VL) assisted intubation training curriculum compared to a traditional direct laryngoscope (DL) assisted curriculum improves neonatal intubation performance of novice intubators in a simulated setting.

**Methods:**

A randomized trial of novice intubators was conducted at the University of Texas-Houston from 6/2013–8/2013. Eligible candidates were randomly assigned to control group (DL curriculum) or intervention group (VL curriculum). Those in the intervention group received instruction with VL videos and practice with Storz C-MAC® VL (Karl Storz, Tuttlingen, Germany) in addition to a traditional curriculum. Intubation performance was evaluated in a simulated setting using a SimNewB® (Laerdal, NY, USA) manikin and traditional intubation equipment. The number of intubation attempts, outcome of each attempt, and time to successful intubation were recorded. The data was analyzed using Fisher's exact test and logistic regression where appropriate.

**Results:**

One hundred twenty-three trainees were enrolled, 62 (50%) in DL group and 61 (50%) in the VL group. Intubation success on first attempt was achieved by 69% (43/62) of the DL group vs. 61% (37/61) of the VL group, P=0.35. Time to successful intubation was 25 sec (interquartile range (IQR) 18, 32) in the DL group and 26.5 sec (IQR 20, 43) in the VL group, P=0.27. Those in the VL group were more likely to need more than two attempts to achieve intubation success, OR=3.09 (95% CI 1.03–9.28).

**Conclusions:**

In a simulated setting, teaching with a VL curriculum did not improve intubation performance compared to teaching with DL. Further studies are needed to determine if VL-based teaching has an impact on clinical intubation performance.

## Introduction

Neonatal endotracheal intubation is a life-saving procedural skill that pediatric trainees are expected to attain competence in performing. Anesthesiologists in training require up to 50 intubation opportunities to achieve a 90% success rate and become competent in performing intubations [1]. In the current training environment, pediatric residents have been reported to have an average of three neonatal intubation opportunities during the course of their residency [2]. Due to changes in delivery room practices, improvement in clinical care, and limitations on the time spent in the Neonatal Intensive Care Unit (NICU), the opportunities to perform endotracheal intubation, specifically in neonatal patients, have become exceedingly limited for pediatric trainees [3-5]. 

Despite the life-saving nature of this procedure and the adverse consequences associated with inadequate performance, best practices on intubation training have been developed from expert opinion, and few empirical studies have examined which technical skills are required and how these skills should be taught [6-7]. Most training programs currently rely on bedside teaching, online training modules, animal labs, or simulation based training to educate their novice intubators.

Video laryngoscopy (VL) is a form of indirect laryngoscopy where visualization of the larynx is performed through the aid of a camera attached to the tip of a laryngoscope blade. VL offers a novel alternative to traditional intubation teaching strategies by allowing the trainee and the instructor to share a view of the airway in real time. It has been shown in simulated settings that trainees using VL and direct laryngoscopy (DL) have similar rates of intubation success [7-8]. However, the role of VL as a teaching aid remains unclear and the high cost associated with the device further warrants rigorous study before recommending widespread use in neonatology. The objective of our study is to determine whether a VL-assisted curriculum, compared to a traditional intubation curriculum that uses DL, improves neonatal intubation performance in a simulated setting.

## Materials and methods

This is a single-center, randomized controlled trial of an educational intervention aimed at novice intubators. The study was conducted at the University of Texas at Houston Surgical and Clinical Skills Center from 06/2013–08/2013. Approval was obtained from the institutional review board of University of Texas at Houston.

### Study population

Novice intubators including Pediatric residents, Neonatology fellows and Respiratory Therapy students with previous experience of five or fewer successful neonatal intubations were invited to participate in this study. The study details were presented to all pediatric trainees at the beginning of their NICU rotations as well as at resident noon conferences. Enrollment was voluntary and interested volunteers were given additional information regarding study-related procedures. Trainees with history of five or fewer successful neonatal intubations were considered to be novice intubators for the purpose of this study. Consented study subjects were randomly assigned either to the intervention arm, to receive VL assisted curriculum or the control arm, to receive DL intubation curriculum. Block randomization method, with a block size of 10, was used to ensure that all groups undergoing the training sessions at any given time were similar in size. Once randomized, participants stayed in the assigned group for the remainder of the study period.

### Study procedures

Study participants were asked to complete a brief survey that prompted them to describe demographic information such as residency class, year of training, and subspecialty as well as their previous experience with intubations. All subjects participated in their assigned intubation curriculum and completed the following three components. First, a didactic session was conducted using a pre-recorded power-point presentation and covered the basics of neonatal airway management, indications for endotracheal intubation, equipment set up, and intubation technique. While all participants viewed the same basic presentation, those randomized to VL curriculum viewed an additional segment that included VL-derived intubation footage from previously performed patient intubations. Participants then attended a skills session where they were given approximately 15 minutes to practice the skills and techniques that were outlined in the presentation. Newborn intubation heads and intubation equipment were made available for this practice session. An expert intubator was present during these sessions to assist with equipment use and to provide coaching. Each participant was allowed to practice their intubation skills at their own discretion, without any restriction on number of attempts or successful intubations during the 15-minute window of time. Those in the video laryngoscopy group had access to a Storz C-MAC VL (Tuttlingen, Germany) and DL to use at their discretion. Participants then moved to the next station for an evaluation session where they were asked to intubate a SimNewB® manikin (Laerdal, NY, USA ) using DL. The number of intubation attempts, outcome of each attempt, and the time required for successful intubation were recorded. Successful intubation was defined as passage of the endotracheal tube into the trachea, as confirmed by the simulator. A maximum of five attempts were allowed by each subject and those unable to intubate successfully within these attempts were designated as unsuccessful intubations. 

### Outcomes of interest

The primary outcome of the study was intubation success on first attempt (yes/no). Secondary outcomes included the time to successful intubation (time from first introduction of the laryngoscope into the manikin's mouth to the passage of the endotracheal tube into the trachea) and number of attempts required to achieve successful intubation of the SimNewB® manikin.

### Data analysis

In order to detect a 30% difference in intubation success between the two study groups, we needed 49 subjects per group and a sample size of 98. Due to abundant interest in the study, we allowed volunteers to enroll in our study beyond the planned sample size since subjects in both study arms would receive a potentially beneficial training experience. Data was analyzed using Fisher's exact test, linear, logistic and Poisson regressions where appropriate. Odds ratios were calculated to compare the odds of intubation success with exposure to VL curriculum vs. exposure to DL curriculum. The data was analyzed using Stata 13.0 (StataCorp LP, TX, USA).

## Results

A total of 123 subjects voluntarily participated in the study and 62 were randomized to the control or DL group and 61 to the intervention or VL group. There were no significant differences in the baseline characteristics such as gender, subspecialty, and residency class between the two study groups [Table 1]. The majority of the study participants were respiratory therapy students and pediatric trainees, with respiratory therapy students being 47% (29/62) of the control group and 49% (30/61) of the intervention group, P=0.86. The control and intervention groups were also comparable in the proportion of subjects with prior patient intubation experience (41% in control group vs. 28% in intervention group, P=0.13) and prior VL experience (11% in control group vs. 18% in intervention group, P=0.32).

**Table 1:** Baseline characteristics of the study population Baseline characteristics of study subjects in control (DL) and intervention (VL) groups were compared using chi-squared analysis.

Characteristic of Interest	Control (DL) (n=62)	Intervention (VL) (n=61)	P-value
Female - n(%)	55 (89)	49 (80)	0.22
Subspecialty of trainee - n(%)			0.86
General pediatrics	26 (42)	27 (44)	
Medicine-Pediatrics	4 (6)	3 (5)	
Pediatrics-Neurology	1 (2)	1 (2)	
Neonatology	2 (3)	0 (0)	
Respiratory Therapy	29 (47)	30 (49)	
Residency class of trainee - n(%)			0.93
Post Graduate Year 1	10 (30)	11 (36)	
Post Graduate Year 2	11 (33)	10 (32)	
Post Graduate Year 3	9 (27)	9 (29)	
Post Graduate Year 4	3 (9)	1 (3)	
Prior intubation experience: yes/no - n(%)	26 (42)	17 (28)	0.13
Prior video laryngoscopy experience: yes/no - n(%)	7 (11)	11 (18)	0.32

Study participants exposed to VL curriculum were less likely to be successful on their first intubation attempt, compared to the control group but this was not statistically significant, OR = 0.68 (95% CI: 0.32, 1.43). Intubation success on first attempt was achieved by 69% (43/62) of the DL group vs. 61% (37/61) of the VL group, P=0.35. In addition, the number of intubation attempts necessary to achieve success was not significantly different between the study groups, OR = 1.71 (95% CI: 0.83, 3.55) [Table 2]. Those in the VL group were more likely to need greater than two attempts to achieve intubation success, OR=3.09 (95% CI 1.03-9.28). Time to successful intubation was not significantly different between the groups, 25 sec (IQR 18, 32 sec) in the DL group vs. 26.5 sec (IQR 20, 43 sec) in the VL group, P=0.27 [Table 3].

**Table 2:** Likelihood of intubation success among study subjects Odds ratios for intubation success and intubation attempts in the VL exposed group (intervention) vs. DL exposed group (control) were calculated using logistic and Poisson regression. Results are stratified by the subject’s level of training.

Outcome of Interest (video laryngoscopy vs. direct laryngoscopy)	Odds Ratio	95%CI	P-value
Intubation success on first attempt	0.68	0.32, 1.43	0.35
Post Graduate Year 1	2.72	0.81, 9.09	0.10
Post Graduate Year 2	0.58	0.21, 1.56	0.29
Post Graduate Year 3	2.24	0.65, 7.64	0.20
Post Graduate Year 4	1.92	0.19, 19.56	0.58
Respiratory Therapy	1.57	0.93, 2.64	0.09
Number of intubation attempts	1.71	0.83, 3.55	0.15

**Table 3:** Comparison of intubation success and time to successful intubation in the control and intervention groups Proportion of study subjects who were successful after each intubation attempt in the VL and DL groups were compared using chi-squared analysis. Time to successful intubation in the VL and DL groups was compared using the Mann-Whitney test.

Intubation outcome	Control (DL) (n=62)	Intervention (VL) (n=61)	P-value
Success on 1^st^ attempt – n (%)	43 (69)	37 (61)	0.35
Success on 2^nd^ attempt – n (%)	14 (23)	11 (18)	0.19
Success on 3^rd^-5^th^ attempts – n (%)	4 (6)	10 (16)	0.10
Unsuccessful after 5 attempts – n (%)	1 (2)	3 (5)	0.37
Time to successful intubation in seconds – Median (IQR)	25.0 (18.0, 32.0)	26.5 (20.0, 43.0)	0.27

## Discussion

We found no significant improvement in trainee intubation performance in the simulated setting when comparing a VL-derived curriculum to a DL-intubation curriculum. Although not statistically significant and the effect size is small, the likelihood of intubation success as well as the number of attempts and the time required for successful intubation were better among the subjects exposed to a DL-intubation curriculum.

Overall, subjects in both groups required more than 20 seconds to perform a successful intubation, which is the time frame recommended by the Neonatal Resuscitation Program. Similar findings have been reported by previous studies that evaluated the utility of VL in teaching neonatal intubation. When a VL system called GlideScope® (Verathon Inc, WA, USA) was studied, none of the study participants were able to intubate successfully within 20 seconds [9]. The reported times in their study are similar to values observed in ours and are concerning as we expect simulated intubations to take less time than the ones done in the clinical setting.

Our study is distinct from the body of literature available on neonatal VL in several important ways. The randomized controlled trial study design is rarely used for educational interventions and by using it we were able to ensure that our study groups were comparable in important baseline characteristics and that our intervention was studied in a rigorous fashion. Our choice of VL equipment is unique as many of the previous studies have used GlideScope® brand of VL. After examining the existing VL market, we realized that the intubation experience varies notably depending on the brand of equipment in use. We chose the Storz® CMAC brand as it resembled the direct laryngoscope more than the other VLs and would therefore limit the challenges associated with translating skills learned via VL to DL. Furthermore, our intervention utilized VL as an educational tool rather than a direct intervention. We realize that the cost associated with VL does not permit routine use of the device in all delivery rooms. And it is important for trainees to be comfortable with equipment they will encounter during real world intubation experiences rather than the VL equipment that may only be available at certain centers. With this intent, we designed a curriculum that would aim to teach sustainable and translatable skills that can be utilized in the post-training, real world setting. We also evaluated our subjects’ performance using traditional equipment in order to assess the impact of our training curriculum on DL-based intubation procedures. Finally, our subject pool was also unique in that we included respiratory therapy students and pediatric trainees of varying specialties. This composition of novice intubators enhances the external validity of our results since an intubation curriculum can be potentially beneficial to all providers who perform procedures in the NICU.

Our study has several important limitations, including the simulated nature of the setting in which intubation skills were taught and evaluated. While we used equipment and airway models that mimic the real world experience as much as possible, we could not simulate factors such as airway secretions that might impair the view of the glottis or bradycardia/desaturations that might limit a provider’s ability to perform the procedure. It is unclear at this time how simulation-based interventions such as these correlate with real world procedural outcomes. The hypothetical advantage of teaching with VL is the ability to enhance the view of the airway, thereby improving the likelihood of proper endotracheal tube placement. However, there may be factors related to VL use that alter the learning process. Since the VL is designed to perform intubation while viewing the airway on a screen, it requires a different set of skills than an intubation done with direct visualization. Therefore, it is possible that while novice intubators may benefit from better familiarity with airway structures, learning the intubation skills using VL may not translate into improvement in intubations done using DL.

In addition, any potential educational benefit gained from improved visualization and recognition of the airway structures may not have been captured by the outcomes we measured in our study and in the setting in which subjects were evaluated. It is unknown whether VL-assisted training might have beneficial effects on patient intubations, rather than the simulated ones done on a manikin. The longer time required to perform successful intubations and the increased number of attempts necessary for successful intubations that we noted in the VL group may be reflective of the transition from using VL to DL equipment. The generalizability of our study results is therefore limited by the simulated nature of our study, and the utility of the VL as a teaching tool may be underappreciated in this setting.

## Conclusions

We did not find an improvement in trainee intubation performance with the use of a VL-assisted intubation curriculum, compared to a standard intubation curriculum that uses traditional equipment. The value of video laryngoscopy as a training aid and its influence on neonatal patient intubations needs to be further explored.

## References

[1] Konrad C, Schupfer G, Wietlisbach M, Gerber H (1998). Learning manual skills in anesthesiology: is there a recommended number of cases for anesthetic procedures?. Anesth Analg.

[2] DeMeo SD, Katakam L, Goldberg RN, Tanaka D (2015). Predicting neonatal intubation competency in trainees. Pediatrics.

[3] Leone TA, Rich W, Finer NN (2005). Neonatal intubation: success of pediatric trainees. J Pediatr.

[4] Bismilla Z, Finan E, McNamara PJ (2010). Failure of pediatric and neonatal trainees to meet Canadian Neonatal Resuscitation Program standards for neonatal intubation. J Perinatol.

[5] Falck AJ, Escobedo MB, Baillargeon JG (2003). Proficiency of pediatric residents in performing neonatal endotracheal intubation. Pediatrics.

[6] Donoghue AJ, Ades AM, Nishisaki A, Deutsch ES (2013). Videolaryngoscopy versus direct laryngoscopy in simulated pediatric intubation. Ann Emerg Med.

[7] Fonte M, Oulego-Erroz I, Nadkarni L, Sánchez-Santos L, Iglesias-Vásquez A, Rodríguez-Núñez A (2011). A randomized comparison of the GlideScope videolaryngoscope to the standard laryngoscopy for intubation by pediatric residents in simulated easy and difficult infant airway scenarios. Pediatr Emerg Care.

[8] Rodriguez-Nunez A, Oulego-Erroz I, Perez-Gay L, Cortinas-Diaz J (2010). Comparison of the GlideScope Videolaryngoscope to the standard Macintosh for intubation by pediatric residents in simulated child airway scenarios. Pediatr Emerg Care.

[9] Fiadjoe JE, Gurnaney H, Dalesio N (2012). A prospective randomized equivalence trial of the GlideScope Cobalt® video laryngoscope to traditional direct laryngoscopy in neonates and infants. Anesthesiology.

